# Antimicrobial susceptibility and genetic characteristics of *Neisseria gonorrhoeae* isolates from Vietnam, 2011

**DOI:** 10.1186/1471-2334-13-40

**Published:** 2013-01-25

**Authors:** Birgitta Olsen, Pham Thi Lan, Daniel Golparian, Emma Johansson, Tran Hau Khang, Magnus Unemo

**Affiliations:** 1 School of Health and Medical Sciences, Örebro University, Örebro, Sweden; 2 Department of Dermato-Venerology, Hanoi Medical University; and National Hospital of Dermatology and Venerology, Hanoi, Vietnam; 3 WHO Collaborating Centre for Gonorrhoea and Other STIs, National Reference Laboratory for Pathogenic Neisseria, Department of Laboratory Medicine, Clinical Microbiology, Örebro University Hospital, Örebro SE-701 85, Sweden

**Keywords:** *Neisseria gonorrhoeae*, Gonorrhoea, Vietnam, Antimicrobial resistance, Extended-spectrum cephalosporins (ESCs), Ceftriaxone, Cefixime, Resistance determinants, *penA*, NG-MAST

## Abstract

**Background:**

Antimicrobial resistance (AMR) in *Neisseria gonorrhoeae* is a major public health concern worldwide. In Vietnam, knowledge regarding *N. gonorrhoeae* prevalence and AMR is limited, and data concerning genetic characteristics of *N. gonorrhoeae* is totally lacking. Herein, we investigated the phenotypic AMR (previous, current and possible future treatment options), genetic resistance determinants for extended-spectrum cephalosporins (ESCs), and genotypic distribution of *N. gonorrhoeae* isolated in 2011 in Hanoi, Vietnam.

**Methods:**

*N. gonorrhoeae* isolates from Hanoi, Vietnam isolated in 2011 (n = 108) were examined using antibiograms (Etest for 10 antimicrobials), *Neisseria gonorrhoeae* multi-antigen sequence typing (NG-MAST), and sequencing of ESC resistance determinants (*penA*, *mtrR* and *penB*).

**Results:**

The levels of in vitro resistance were as follows: ciprofloxacin 98%, tetracycline 82%, penicillin G 48%, azithromycin 11%, ceftriaxone 5%, cefixime 1%, and spectinomycin 0%. The MICs of gentamicin (0.023-6 mg/L), ertapenem (0.002-0.125 mg/L) and solithromycin (<0.016-0.25 mg/L) were relatively low. No *penA* mosaic alleles were found, however, 78% of the isolates contained an alteration of amino acid A501 (A501V (44%) and A501T (34%)) in the encoded penicillin-binding protein 2. A single nucleotide (A) deletion in the inverted repeat of the promoter region of the *mtrR* gene and amino acid alterations in MtrR was observed in 91% and 94% of the isolates, respectively. *penB* resistance determinants were detected in 87% of the isolates. Seventy-five different NG-MAST STs were identified, of which 59 STs have not been previously described.

**Conclusions:**

In Vietnam, the highly diversified gonococcal population displayed high in vitro resistance to antimicrobials previously recommended for gonorrhoea treatment (with exception of spectinomycin), but resistance also to the currently recommended ESCs were found. Nevertheless, the MICs of three potential future treatment options were low. It is essential to strengthen the diagnostics, case reporting, and epidemiologic surveillance of gonorrhoea in Vietnam. Furthermore, the surveillance of gonococcal AMR and gonorrhoea treatment failures is imperative to reinforce. Research regarding novel antimicrobial treatment strategies (e.g., combination therapy) and new antimicrobials is crucial for future treatment of gonorrhoea.

## Background

Infections caused by *Neisseria gonorrhoeae* are major public health problems globally. In 2008, the World Health Organization (WHO) estimated 106 million new cases of gonorrhoea among adults worldwide. This places gonorrhoea as the most common bacterial sexually transmitted infection (STI), that is, together with *Chlamydia trachomatis* infections also estimated to 106 million cases. The highest gonorrhoea incidence was in the WHO Western Pacific region (WPR), estimated to 42 million cases [1]. In general, the highest rates of gonorrhoea have been found in developing countries and especially in lower socio-economic groups, men who have sex with men (MSM), commercial sex workers (CSWs) and their clients [1,2].

*N. gonorrhoeae* has developed resistance to all antimicrobials previously recommended as first-line treatment of gonorrhoea, e.g. penicillins, tetracyclines and fluoroquinolones, as well as macrolides such as erythromycin and azithromycin. Extended-spectrum cephalosporins (ESCs) are currently the recommended first-line antimicrobials in most countries worldwide. However, recent two decades in vitro resistance also to ESCs have emerged and spread [2-6]. Verified treatment failures with the oral ESC cefixime have been reported in Japan and recently in several European countries [6-12]. With the injectable ESC ceftriaxone, a few cases of confirmed treatment failure of pharyngeal gonorrhoea have been reported [13-16] and most worryingly the first three extensively-drug resistant (XDR) [5] gonococcal strains with high-level resistance to ceftriaxone have been described [9,16,17]. Ceftriaxone is also the last remaining option for empirical first-line antimicrobial monotherapy of gonorrhoea. In this developing situation, including the fear that gonorrhoea may become untreatable, the WHO [18], European Centre for Disease Prevention and Control (ECDC) [19] and Centers for Disease Control and Prevention (CDC), USA [20] have published action/response plans to combat and mitigate the widespread of multidrug-resistant gonorrhoea. However, even if these action/response plans will be fully implemented ultimately it is vital to develop new treatment strategies and particularly novel antimicrobials. Gentamicin, ertapenem and solithromycin have been previously investigated and may be effective treatment alternatives, in antimicrobial monotherapy and particularly in combination therapy [6,21-25].

Mutations in the *penA* gene encoding the penicillin-binding protein 2 (PBP2) is the main determinant for decreased susceptibility and resistance to ESCs. Acquisition of a *penA* mosaic gene or an alteration of amino acid A501 in PBP2 result in a lower affinity for ESCs and consequently a decreased ESC susceptibility [6,9,16,26-32]. Mutations in the promoter or coding sequence of the repressor gene *mtrR* cause over-expression of the MtrCDE efflux pump system that export the ESCs out from the cell. This further decreases the susceptibility to ESCs [6,9,16,28,32-35]. Alterations of amino acid G101 and A102 in the porin PorB1b (the *penB* resistance determinant), which is encoded by the *porB1b* gene, result in decreased permeability and further decreased susceptibility to ESCs [6,9,16,28,32,34,36,37]. There is still at least one unknown non-transformable ESC resistance determinant [6,9,16,32].

Historically, antimicrobial resistance (AMR) in *N. gonorrhoeae* appears to mostly have emerged in WHO WPR and subsequently spread globally, by sex tourists and travellers [2,4-6]. In WHO WPR, Vietnam is the easternmost country on the Indochina Peninsula in Southeast Asia; bordering to China, Laos, and Cambodia and with an estimated population of about 90 millions. In Vietnam, for treatment of gonorrhoea ceftriaxone 250 mg × 1 intramuscularly is the recommended first-line and cefixime 400 mg × 1 orally the recommended second-line. However, a wide variety of antimicrobials such as penicillins, fluoroquinolones, macrolides, and spectinomycin may still be used for treatment. Worryingly, the knowledge regarding *N. gonorrhoeae* AMR in Vietnam is limited, and data concerning genetic characteristics (molecular epidemiology and genetic resistance determinants) is totally lacking. Regarding prevalence of gonorrhoea in Vietnam, some few studies have showed low prevalences among women of reproductive age [38,39], one study of female sex workers described a prevalence of 14.9% [40] and one study of men who have sex with men reported a prevalence of 1.8% for gonorrhoea and 4.7% for gonorrhoea/chlamydia [41]. In general, the gonorrhoea diagnostics in Vietnam is suboptimal and individuals commonly prefer private healthcare providers, self-medication or treatment by drug sellers instead of accessing public services [38,42]. Accordingly, no reliable national incidence data in regard to bacterial STIs, including gonorrhoea, exist because the STI diagnostics (quality including availability), case reporting (particularly among private health care providers) and epidemiological surveillance are suboptimal.

The aims of the present study were to investigate the phenotypic AMR (previous, current and possible future treatment options), genetic ESC resistance determinants, and genotypic distribution of *N. gonorrhoeae* isolated in 2011 in Hanoi, Vietnam.

## Methods

### *Neisseria gonorrhoeae* isolates

All 108 *N. gonorrhoeae* isolates were obtained at the National Hospital of Dermatology and Venerology, Hanoi, Vietnam from January to September 2011. The isolates were cultured from consecutive symptomatic gonorrhoea patients; 19 females, 85 males, and four patients lacking data of sex and age. Mean age for the females was 28.5 years (median age: 26.5 years; range: 6 to 40 years) and for the males 29 years (median age: 27 years; range: 18 to 60 years). Of the patients, 72% were from Hanoi, 18% from other Red river delta provinces, 6% from different other provinces, and for 4% no information regarding place of accommodation was available.

All isolates were lyophilized and sent to the WHO Collaborating Centre for Gonorrhoea and Other STIs, Örebro University Hospital, Sweden, for species confirmation by culture on selective agar media, a sugar utilization test and the PhadeBact GC Monoclonal test (Bactus AB, Solna, Sweden), and subsequently preserved as previously described [43]. All examined gonococcal isolates were cultured and stored as part of the routine diagnostics (standard care) and no patient identification information was used in the study.

### Antimicrobial susceptibility testing

The minimum inhibitory concentration (MIC; mg/L) of cefixime, ceftriaxone, penicillin G, azithromycin, ciprofloxacin, spectinomycin, tetracycline, gentamicin, and ertapenem were analysed using the Etest method (bioMerieux AB, Solna, Sweden), according to the instructions from the manufacturer, and to solithromycin with agar dilution method, in accordance with the Clinical and Laboratory Standards Institute (CLSI (M100-S22)). All results were interpreted using whole MIC dilutions and where available, breakpoints for susceptibility (S) and resistance (R) according to The European Committee on Antimicrobial Susceptibility Testing (EUCAST [http://www.eucast.org]) were used (Table 1). For gentamicin, ertapenem and solithromycin, no breakpoints are stated by any organization.

**Table 1:** **Antimicrobial susceptibility of 108 *Neisseria gonorrhoeae *isolates from Hanoi, Vietnam in 2011**

**Antimicrobial (Breakpoints (mg/L))**	**Susceptible no. (%)**	**Intermediate no. (%)**	**Resistant no. (%)**
**Ciprofloxacin **(S ≤ 0.032, I = 0.064, R > 0.064)*	2 (2)	0	106 (98)
**Tetracycline **(S ≤ 0.5, I = 1.0, R > 1.0)*	7 (6)	13 (12)	88 (82)
**Penicillin G **(S ≤ 0.064, I = 0.125-1.0, R > 1.0)*	2 (2)	54 (50)	52 (48)
**Azithromycin **(S ≤ 0.25, I = 0.5, R > 0.5)*	67 (62)	29 (27)	12 (11)
**Ceftriaxone **(S ≤ 0.125, I = NA, R > 0.125)*	103 (95)	NA	5 (5)
**Cefixime **(S ≤ 0.125, I = NA, R > 0.125)*	107 (99)	NA	1 (1)
**Spectinomycin **(S ≤ 64, I = NA, R > 64)*	108 (100)	NA	0
	**MIC range (mg/L)**	**MIC**_**50 **_**(mg/L)**	**MIC**_**90 **_**(mg/L)**
Gentamicin**	0.032–8	4	4
Ertapenem**	0.002–0.125	0.012	0.032
Solithromycin**	<0.016-0.25	0.064	0.125

NA, not applicable.

* Breakpoints for susceptible (S ≤ x mg/L) and resistant (R > y mg/L) according to The European Committee on Antimicrobial Susceptibility Testing (EUCAST; http://www.eucast.org).

** Breakpoints not stated by any organization.

### Isolation of genomic DNA

Bacterial DNA was isolated in the robotized NorDiag Bullet instrument (NorDiag ASA Company, Oslo, Norway) using the BUGS’n BEADS™ STI-*fast* kit (NorDiag ASA Company, Oslo, Norway), according to the instructions from the manufacturer.

### Molecular epidemiological typing

*N. gonorrhoeae* multiantigen sequence typing (NG-MAST) was performed as previously described [44,45]. NG-MAST allele numbers of the more variable segments of *porB* and *tbpB*, and sequence types (STs) were assigned using the NG-MAST website (http://www.ng-mast.net).

### Sequencing of genetic ESC resistance determinants

Detection of ESC resistance determinants in *N. gonorrhoeae*, i.e. *penA* mosaic alleles, alterations of A501 in PBP2, mutations in the promoter and/or coding sequence of the *mtrR* gene, and the *penB* resistance determinant, were performed by sequencing as previously described [34,45].

### Sequence alignments

Multiple-sequence alignments of nucleotide sequences and the deduced corresponding amino acid sequences were performed in the software BioEdit Sequence Alignment Editor version 7.0.9.0 with manual adjustment.

## Results

### Antimicrobial susceptibility

The antimicrobial susceptibility of all isolates are summarised in Table 1.

Briefly, the levels of in vitro resistance were as follows: ciprofloxacin 98%, tetracycline 82%, penicillin G 48%, azithromycin 11%, ceftriaxone 5%, cefixime 1%, and spectinomycin 0%. For gentamicin (MIC range: 0.023–6 mg/L), ertapenem (0.002–0.125 mg/L) and solithromycin (<0.016–0.25 mg/L) there are no breakpoints stated by any organization, however, the MICs were relatively low, indicating a susceptible gonococcal population (Table 1).

According to the breakpoints stated by the EUCAST, five (5%) isolates displayed in vitro resistance to ceftriaxone (all having MIC = 0.25 mg/L) and 1 (1%) to cefixime (MIC = 0.25 mg/L). However, in Japan also gonococcal isolates with cefixime MIC of 0.125 mg/L have resulted in treatment failure with cefixime (200 mg × 2) [7]. Accordingly, isolates with an ESC MIC of 0.125 mg/L might be considered as having at least an in vitro decreased susceptibility to ESCs. In the present study, the number of isolates with an ESC MIC ≥ 0.125 mg/L were high, i.e., 30 (28%) and 9 (8%) for ceftriaxone and cefixime, respectively.

### ESC genetic resistance determinants (*penA*, *mtrR* and *penB*)

The NG-MAST STs, presence of ESC resistance determinants, and MICs of ESCs among all the isolates with in vitro resistance or decreased susceptibility to ESCs (MIC ≥ 0.125 mg/L; n = 30) are summarized in Table 2.

**Table 2:** ***Neisseria gonorrhoeae *with decreased susceptibility or resistance to cefixime or ceftriaxone (≥0.125) isolated in Hanoi, Vietnam in 2011 (n = 30)**

**NG-MAST**	**Minimum inhibitory concentration**		***penA *alteration**	***mtrR *alteration**		**PorB1b (*penB *alteration)**	
	**Cefixime**	**Ceftriaxone**	**A501 alteration (*penA *allele [**[16]**])**	**A-deletion in promoter region**	**MtrR coding region**	**G101**	**A102**
ST4787 (n = 1)	0.25	0.25	T (XVIII)	Yes	A39T	K	D
ST4787 (n = 2)	0.125	0.25	T (XVIII)	Yes	A39T	K	D
ST4787 (n = 1)	0.125	0.125	V (A501 New**)	Yes	H105Y	K	D
ST4787 (n = 3)	0.064	0.125	T (XVIII)	Yes	A39T	K	D
ST4787 (n = 1)	0.032	0.125	T (XVIII)	Yes	A39T	K	D
ST7159 (n = 2)	0.064	0.125/0.25	T (XVIII)	Yes	D79N T86A H105Y	K	D
ST4676 (n = 1)	0.125	0.25	T (XVIII)	Yes	H105Y	K	G
3 STs^a ^(n = 4)	0.125	0.125	T (XVIII)	Yes	A39T	K/D^b^	D/G^b^
12 STs (n = 15)	0.064	0.125	T/V/WT^c^	Yes	A39T G45D H105Y^d^	K/D/T^e^	D/G/WT^e^

NG-MAST, *Neisseria gonorrhoeae *multiantigen sequence typing; ST, sequence type.

^a ^ST7739 (n = 2), ST7160 (n = 1) and ST7741 (n = 1).

^b ^G101D/A102G (n = 2) and G101K/A102D (n = 1).

^c ^A501V New** (n = 9), XVIII (n = 3), XIII (n = 2) and XII (n = 1).

^d ^H105Y (n = 8), A39T (n = 4), and G45D (n = 3).

^e ^G101K/A102D (n = 11), G101D/A102G (n = 2), G101D/WT (n = 1), G101T/A102D (n = 1).

None of the 30 isolates with in vitro resistance or decreased susceptibility (MIC ≥ 0.125 mg/L) to ceftriaxone (n = 30) and cefixime (n = 9) contained a *penA* mosaic allele. However, 17 (57%) of those 30 isolates contained an A501T alteration and 12 (40%) had an A501V alteration in PBP2, and all 30 isolates contained *mtrR* and *penB* resistance determinants (Table 2).

Moreover, in the entire material (108 isolates) no isolates containing a *penA* mosaic allele were found. However, 47 (44%) of the isolates contained a PBP2 A501V alteration and 37 (34%) a PBP2 A501T alteration. The alleles, according to the previously published numbering of PBP2 amino acid sequences in *N. gonorrhoeae*[16], were PBP allele XVIII (n = 37), A501V New allele (n = 28), XIII (n = 13), XXI (n = 3), XXXIII (n = 2), and A501V New allele (n = 1). The MICs of cefixime and ceftriaxone for the isolates with PBP2 A501 alterations ranged from <0.016–0.25 mg/L (mean MIC: 0.064 mg/L) and 0.016–0.25 mg/L (mean MIC: 0.064 mg/L), respectively.

A single nucleotide (A) deletion in the inverted repeat of the promoter region of the *mtrR* gene was observed in 98 (91%) of the 108 isolates. Amino acid alterations in MtrR were found in 102 (94%) isolates. Accordingly, 45 (42%) had alterations in amino acid residue H105, 30 (28%) in A39, and 27 (25%) in G45. Furthermore, the recently described C-to-T transition 120 bp upstream of the *mtrC* start codon (termed *mtr*120) that results in a consensus −10 element, a novel promoter for *mtrCDE* transcription and accordingly increased expression of the MtrC-MtrD-MtrE efflux pump was found in four (4%) isolates [35].

Alterations in amino acid residues G101 and/or A102 of PorB1b (*penB* resistance determinant) were detected in 94 (87%) of the isolates. All those isolates possessed an altered G101, and 84 (78%) contained an additional alteration of A102.

### Molecular epidemiological characterisation

The 108 isolates were assigned to 75 different NG-MAST STs. Fifty-nine (79%) of these STs have not been previously described. The most prevalent ST was ST4787 (n = 11), followed by ST7720 (n = 5), ST7741 (n = 4), ST7724 (n = 3), and ST7155 (n = 3). Of those STs, all except ST4787 were novel STs. Twelve STs contained two isolates and 58 STs were represented by single isolates (Table 3).

**Table 3:** **Minimum inhibitory concentrations (MICs, mg/L) of cefixime and ceftriaxone for *Neisseria gonorrhoeae *(n = 108) isolated in Hanoi, Vietnam in 2011**

**NG-MAST ST (No. of isolates)**	**Anti-microbial**	**No. of isolates with MIC (mg/L):**				
		**≤0.016**	**0.032**	**0.064**	**0.125**	**0.25**
4787 (11)	IX		3	4	3	1
	TX			3	5	3
7720 (5)^*a*^	IX	1	4			
	TX		5			
7741 (4)^*a*^	IX			3	1	
	TX				4	
7155 (3)^a^	IX		1	2		
	TX		2	1		
7724 (3)^*a*^	IX		3			
	TX			3		
3493 (2)	IX	2				
	TX	2				
7139 (2)^*a*^	IX	2				
	TX	2				
7140 (2)^*a*^	IX		1	1		
	TX		2			
7159 (2)^*a*^	IX			2		
	TX				1	1
7718 (2)^*a*^	IX			2		
	TX				2	
7722 (2)^*a*^	IX	2				
	TX		2			
7731 (2)^*a*^	IX		2			
	TX		2			
7734 (2)^*a*^	IX			2		
	TX			1	1	
7735 (2)^*a*^	IX		2			
	TX		1	1		
7736 (2)^*a*^	IX		1	1		
	TX			2		
7739 (2)^*a*^	IX				2	
	TX				2	
7748 (2)^*a*^	IX		1	1		
	TX		1	1		
Unique STs (58)^*b*^	IX	15	16	25	2	
	TX	12	15	20	10	1

NG-MAST, *Neisseria gonorrhoeae *multiantigen sequence typing; ST, sequence type;

MIC, minimum inhibitory concentration; IX, cefixime; TX, ceftriaxone.

^*a*^New sequence types found in the present study.

^*b*^Unique STs represented by single isolates.

Eight (73%) of the ST4787 isolates (n = 11) displayed in vitro resistance or decreased susceptibility to ceftriaxone (MIC ≥ 0.125 mg/L) and four (36%) of them resistance or decreased susceptibility to cefixime (MIC ≥ 0.125 mg/L). Ten (91%) of the ST4787 isolates contained an identical A501T altered PBP2 allele (XVIII), a single nucleotide (A) deletion in the *mtrR* promoter, A39T alteration in MtrR and *penB* (G101K/A102D) (Table 2).

## Discussion

In the present study, the susceptibility to previous, current and possible future antimicrobial treatment options in *N. gonorrhoeae* isolated in 2011 in Vietnam were studied. Exceedingly high prevalence of resistance was observed for previous first-line antimicrobials such as ciprofloxacin (98%), tetracycline (82%) and penicillin G (48%), but also relatively high for azithromycin (11%) (Table 1). This is in accordance with previous studies from other countries in WHO WPR such as Japan, The Philippines, China, Hong Kong, Korea and Taiwan [4,46-51], South Asia, e.g., India, Pakistan, Thailand, Sri Lanka and Bhutan [47,52,53], and many other regions globally [3-6,18,19,54-58]. None of these antimicrobials can be recommended for first-line empiric therapy of gonorrhoea in Vietnam as well as in most parts of the world.

Most worryingly, 5% of the gonococcal isolates in Vietnam displayed in vitro resistance to ceftriaxone and 1% to cefixime (Table 1). Furthermore, in total 30 (28%) and 9 (8%) displayed in vitro resistance or decreased susceptibility (MIC ≥ 0.125 mg/L) to ceftriaxone and cefixime, respectively (Table 2). All except one of these 30 isolates contained an A501 alteration in PBP2, and all 30 comprised *mtrR* and *penB* resistance determinants. All these resistance determinants have previously been reported as associated with decreased susceptibility and resistance to ESCs [3,5,6,9-11,14-17,25-32,34,51,58,59]. However, in Vietnam no isolates with a mosaic PBP allele, which has been strongly associated with decreased susceptibility and resistance to ESCs in many countries, was found [3,5,6,31,32,58]. The predominance of A501-altered *penA* alleles in a gonococcal population with in vitro resistance and decreased susceptibility to ESCs as observed in Vietnam has also been previously described in publications from other countries such as Korea [28] and Australia [59,60]. In the present study, the MICs of cefixime and ceftriaxone for the isolates with PBP2 A501 alterations ranged from <0.016–0.25 mg/L and 0.016–0.25 mg/L, respectively. This large variety in the MICs of ESCs among these isolates could not be explained by the presence or absence of other resistance determinants such as *mtrR* and *penB*. The reasons for the highly variable ESC MICs of isolates with A501-altered PBP2 remain unknown, but possibly the isolates with higher ESC MICs also contain the unknown non-transformable penicillin and cephalosporin resistance determinant “factor X” [6,9,16,32]. Accordingly, detection of the currently known ESC genetic resistance determinants (*penA* mosaic allele or alteration of A501 in PBP2, *mtrR* and *penB*) does not strictly reflect the exact MICs of ESCs and, accordingly, cannot replace traditional culture-based AMR testing (which needs to be strengthened worldwide) or be used in the management of clinical patients. However, detection of genetic resistance determinants combined with molecular epidemiological typing (NG-MAST) can still be valuable for surveillance purposes, i.e. to monitor the emergence and spread of isolates with decreased susceptibility or resistance to ESCs, enhancing our knowledge regarding the effects on ESC MICs of different *penA* alleles, etc. The present study shows that the prevalence of gonococcal isolates with phenotypic and genetic resistance or decreased susceptibility to ESCs in Vietnam is high. Longitudinal studies in Vietnam examining the ESC MICs over time supplemented by determination of genetic resistance determinants would be exceedingly valuable.

Disquietingly, despite full implementation of the action/response plans recently launched [18-20], ultimately new treatment strategies and particularly novel antimicrobials are essential to develop. In the United Kingdom [61], Europe [62] and USA [63], the recently revised treatment guidelines all recommend dual antimicrobial therapy (mainly with ceftriaxone plus azithromycin). However, in vitro and in vivo resistance to both ceftriaxone and azithromycin have already been verified and dual antimicrobial therapy may not be feasible and/or affordable in all less-resourced settings. Accordingly, new antimicrobials for treatment of gonorrhoea are essential to develop. There are few new compounds in sight [3,6,16,64]. The new fluoroketolide solithromycin (class: macrolides) has recently been investigated and showed an activity superior to that of most other antimicrobials previously or currently recommended for treatment of gonorrhoea [23]. In the present study, despite that 11% (27%) of the isolates were resistant (intermediate resistance) to azithromycin the MIC range of solithromycin was only <0.016–0.25 mg/L and MIC_90_ was 0.125 mg/L. Accordingly, these results further support solithromycin as a possible future option for single and particularly dual antimicrobial therapy of gonorrhoea. Ertapenem, a parenteral 1-β-methyl-carbapenem, has in a previous study shown advantages over ceftriaxone for ceftriaxone-resistant isolates [25]. In the present study, all 30 isolates with in vitro resistance (n = 5) and decreased susceptibility (n = 25) to ceftriaxone displayed ertapenem MIC values ranging from 0.016 mg/L to 0.032 mg/L (Table 2), and the MIC_90_ for all isolates was 0.032 mg/L. Finally, the aminoglycoside gentamicin has been used for nearly two decades in Malawi to treat gonorrhoea (mainly in syndromic management together with doxycycline), with a remained high in vitro susceptibility in the gonococcal populations [21,24]. An evaluation of gentamicin in vitro susceptibility of *N. gonorrhoeae* isolates in Europe showed that 95% of 1366 isolates were distributed within a narrow MIC range of 4–8 mg/L [65]. In the present study, the MIC range was 0.032–6 mg/L and MIC_90_ was 4 mg/L. There are yet no international interpretative criteria for MICs of solithromycin, ertapenem or gentamicin. However, in studies from Malawi breakpoints have been suggested, that is, susceptible: MICs ≤ 4 mg/L, intermediate susceptible: MIC = 8–16 mg/L and resistant: MICs ≥ 32 mg/L [21].

Finally, using NG-MAST the present study showed a diversified population of *N. gonorrhoeae* in Hanoi, Vietnam during 2011, with 75 different NG-MAST STs among the 108 isolates. The high number of unique STs (n = 58) and STs that have not been described earlier (n = 59) may be associated with suboptimal diagnostics (only random gonorrhoea patients and/or isolates are identified), contact tracing (sexual contacts having the identical ST are not traced) and epidemiological surveillance (sexual transmission chains spreading a single ST are not identified or followed-up), STs evolved locally in Vietnam (STs are not previously described because no NG-MAST studies have previously been performed in the country) or imported from abroad. However, some minor ST clusters caused by clonal spread of, e.g. ST4787 (n = 11), ST7720 (n = 5) and ST7741 (n = 4) were identified which indicate some sexual transmission chains. Of the eleven ST4787 isolates, eight displayed in vitro resistance or decreased susceptibility to ceftriaxone and all eleven isolates showed A501 alteration in PBP2 as well as contained *mtrR* and *penB* resistance determinants.

## Conclusions

*N. gonorrhoeae* isolated in Vietnam during 2011 showed a high genetic diversity and high levels of in vitro resistance to antimicrobials previously recommended for gonorrhoea treatment, such as ciprofloxacin, tetracycline, penicillin G and azithromycin. Furthermore, 5% (28%) of the isolates were in vitro resistant (had a decreased susceptibility) to ceftriaxone, currently the recommended drug of choice for treatment of gonorrhoea. Nevertheless, no resistance to spectinomycin, which remains available in Vietnam, was found and the MICs of three potential future treatment options were low. Research regarding novel antimicrobial treatment strategies (e.g., combination therapy) and new antimicrobials is crucial for future treatment of gonorrhoea.

Finally, it is of great importance to strengthen the *N. gonorrhoeae* diagnostics, case reporting, and surveillance of epidemiology, AMR as well as gonorrhoea treatment failures in Vietnam.

## Competing interests

The authors declare that they have no competing interests.

## Authors’ contributions

MU and PTL designed the study. BO, DG and EJ performed all the laboratory analysis. All authors analysed and interpreted the data, and were involved in the preparations of the paper. All authors read and approved the final manuscript.

## Pre-publication history

The pre-publication history for this paper can be accessed here:

http://www.biomedcentral.com/1471-2334/13/40/prepub
